# Supplementary material for: Research on dual-waterway cooling system of high-power-density permanent magnet synchronous machine
Source: PLoS One. 2025 Sep 12;20(9):e0332155. doi: 10.1371/journal.pone.0332155 (PMC12431533; doi:10.1371/journal.pone.0332155)
Supplement: Attachment — Submitted filename: Response to Reviewers.doc [file pone.0332155.s002.doc]

**^[1]^Original Manuscript ID:** PONE-D-25-31613

**Original Article Title: “**Research on Dual-Waterway Cooling System of High-Power-Density Permanent Magnet Synchronous Machine”

**To:** PLOS ONE Editor

**Re:** Response to reviewers

Dear Editor,

Thank you for allowing a resubmission of our manuscript, with an opportunity to address the reviewers’ comments.

We are uploading (a) our point-by-point response to the comments (below) (response to reviewers), (b) an updated manuscript with yellow highlighting indicating changes, and (c) a clean updated manuscript without highlights.

We have reviewed and carefully taken the reviewers’ comments into consideration in preparing our correction, which has resulted in a paper that is clearer, more compelling, and broader. We sincerely hope that this revised manuscript has addressed all the comments and suggestions. We appreciate for warm work of reviewers and editor earnestly, and hope that the correction will meet with approval. Thanks again.

Best regards,

<Yingying Xu, Xiaoqian Duan, Mingxia Xu Aochen Han and Wenyu Yu.>

**Reviewer#1, Concern # 1:** The authors need to clarify whether keywords should be included in the main text.

**Author response:** Thank you very much for your advice. We have confirmed that the keywords do not need to be included in the main text, because the journal submission template does not cover this content. We have already submitted the keywords to the corresponding section of the submission system. The keywords are as follows.

Keywords: HPDPMSM, fluid-solid coupling, motor structure, heat dissipation, thermodynamic calculation, temperature distribution

**Reviewer#1, Concern # 2:** The introduction structure requires adjustment. The first and second paragraphs could be merged. Additionally, the authors should consider expanding references related to electric vehicles, such as fuel cell vehicles.

**Author response:** Thanks very much for your comment. We have merged the first and second paragraphs of the introduction section, and added References [6], [7] on fuel cell vehicles to further enhance its relevance to the automotive field. The content of the fuel cell journal which we have added is as follows.

The motor's temperature increase substantially affects its performance and durability, while also indirectly impacting the operational condition of the fuel cell system [6]. Especially in high-load conditions, the motor's temperature escalates rapidly. Excessively high operating temperatures can disrupt the rate of chemical reactions and voltage output in fuel cells, leading to diminished efficiency or unreliable operation due to their sensitivity to temperature fluctuations [7]. This will impact the vehicle's total power performance. Consequently, the thermal dissipation issue of HPDPMSM is examined, highlighting the necessity for an effective cooling system design.

[6] Liu Z, Zhang B, Xu S. Research on Air Mass Flow-Pressure Combined Control and Dynamic Performance of Fuel Cell System for Vehicles Application. Applied Energy, 2022, 309: 118446. doi.org/10.1016/j.apenergy.2021.118446.

[7] Liu Z, Xu S, Zhang B, et al. Voltage Tracking and Regulation of Vehicle PEMFC System Under Low Load Condition Based on Fuzzy LQG Hybrid Strategy. ISA transactions, 2025. doi.org/10.1016/j.isatra.2025.06.008.

We have added the related statements in Introduction (line 39, page 3 to line 46, page 3), and Ref. [6] [7].

**Author action:** We updated the manuscript by merging the first and second paragraphs in Introduction, and adding the related statements (line 39, page 3 to line 46 page 3), Ref. [6] [7].

**Reviewer#1, Concern # 3:** The resolution of all figures should be improved, and vector graphics should be provided.

**Author response:** Thanks for your constructive suggestion. We have enhanced the resolution of all figures to ensure they are clearer. Meanwhile, to better present the data and information, we have provided vector graphics versions of Figs 13A, 15, 16, 17 and 18.

**Author action:** We updated the manuscript by improving the resolution of all figures.

**Reviewer#1, Concern # 4:** For Figure 13, is the "Maximum Temperature Rise" relative to ambient temperature or a specific baseline condition?

**Author response:** Thanks for your constructive suggestion. The maximum temperature rise is relative to ambient temperature in Fig 13. We have added relevant explanations, as follows.

An analysis is conducted on the impact of different coolants on the maximum temperature rise of the stator, rotor, permanent magnet, and winding in relation to an ambient temperature of 300K, as illustrated in Fig 15.

We have added relevant explanations in line 402, page 22 to line 404, page 22.

**Author action:** We updated the manuscript by adding relevant explanations. (line 402, page 22 to line 404, page 22).

**Reviewer#1, Concern # 5:** Key performance indicators for the "N48UH" permanent magnet material should be provided.

**Author response:** Thanks for your constructive suggestion. We have provided the key performance indicators for the N48UH permanent magnetic material in Table 2.

The permanent magnet material adopts the N48UH, which provides a high magnetic field strength for the motor. Table 2 presents the performance parameters of permanent magnets.

Table 2. Performance parameters of permanent magnets

| Parameter | Maximum magnetic energy product (MGOe) | Remanence (kGs) | Magnetic coercivity (KOe) | Intrinsic coercivity  (kOe) | Reversible Temperature Coefficient (%/℃) | | Max Operating Temperature  (℃) |
| --- | --- | --- | --- | --- | --- | --- | --- |
|  |  |  |  |  | \|αBr\| | \|βHcj\| |  |
| N48UH | 47 | 13.8 | 13.3 | 25 | 0.11 | 0.5 | 180 |

We have added the related statements in line 104, page 6 to line 107, page 6.

**Author action:** We updated the manuscript by adding relevant statements. (line 104, page 6 to line 107, page 6).

**Reviewer#1, Concern # 6:** The English language requires further polishing.

**Author response:** Thanks for your constructive suggestion. We have improved the English expression of the entire manuscript to ensure that the language is more fluent and accurate.

**Author action:** We updated the manuscript by improving the English expression.

**Reviewer#2, Concern # 1:** For the keywords, 'heat dissipation', 'thermodynamic calculation', 'temperature distribution', and 'motor structure' should be added to attract a broader readership.

**Author response:** Thank you very much for your kind suggestions. We have added “motor structure, heat dissipation, thermodynamic calculation, and temperature distribution” as keywords. The keywords are as follows.

Keywords: HPDPMSM, fluid-solid coupling, motor structure, heat dissipation, thermodynamic calculation, temperature distribution

**Author action:** We updated the manuscript by adding relevant keywords.

**Reviewer#2, Concern # 2:** The abstract and introduction do not clearly distinguish the innovation boundary between this study and previous works (for example, the dual-channel water cooling structure has been proposed in Ref. [14]), and it is necessary to quantify and compare the improvement ratio of the heat dissipation efficiency of the existing cooling system.

**Author response:** Thanks for your constructive suggestion. The cooling effects discussed in References [11], [16], [17], and [18] have been subjected to detailed quantitative analyses, with the relevant data extensively elaborated upon in the text. Following the inclusion of two additional references in the introduction, the original Reference [14] has been revised to Reference [16]. The specific modifications are as follows.

A unique water-cooling arrangement using cooling tubes within the stator yoke has been proposed to enhance thermal management. This design attained a maximum rotor temperature reduction of 11.17%, and the influence of coolant type and flow rate on heat exchange efficiency was comprehensively examined [11].

Chen, W., et al. examined the structure and environmental influences of the PMSM employed as the propulsion system for underwater UAVs, proposing a dual-channel water-cooled configuration integrated into the motor housing that obviates the necessity for auxiliary devices and promotes internal circulation for efficient heat dissipation within the motor. This method decreased the temperature of the permanent magnet by 12K [16]. A double-cycle arrangement utilizing external liquid cooling and internal air cooling has been developed to address the problem of internal heat dissipation within the motor. A cooling fan was affixed to the slotted stator yoke and spinning shaft to enhance internal airflow and mitigate temperature elevation. The permanent magnet's maximum temperature decreased by 13.5K [17]. Li, Y., et al. developed a motor cooling system comprising an internal oil circulation and an external water circulation, with the latter utilizing a spoiler configuration. This method lowers the winding temperature by 5K compared to water cooling, hence markedly improving the motor's heat dissipation efficiency [18].

We have added relevant statements in Introduction. (line 51, page 3 to line 55, page 4, and line 68, page 4 to line 80, page 5).

**Author action:** We updated the manuscript by adding relevant statements in Introduction. (line 51, page 3 to line 55, page 4, and line 68, page 4 to line 80, page 5).

**Reviewer#2, Concern # 3:** The fluid-solid coupled temperature field model has not been experimentally verified, and only single/double water channel structures are compared through simulation, which is not credible enough. It is recommended to increase test results, provide measured temperature data at key locations for comparison with simulation results, and verify the model error rate.

**Author response:** Thanks for your kind question. We have currently conducted verification solely on the single-waterway configuration of the motor. In the test and simulation, we adopt the same cooling method, namely oil cooling. Fig 12 shows the motor test platform. The test environment temperature is 28℃, and the simulated environment temperature is set at 300K. Fig 13 shows the relevant results. It has been observed that a 5.76K temperature rise discrepancy exists between the test and simulation results, primarily due to the simplification of the motor winding's end during the simulation process. However, the relative error of their results being less than 5% proves the validity of the simulation model, which further proves the rationality of the simulation model of the double waterway. As for the dual-waterway motor, it is still in the production phase. The specific modifications are as follows.

**Experimental facility**

Fig 12 shows the motor test platform. The controller transforms direct current into alternating current to provide power for the motor. The cooling duct delivers coolant to the motor, while the induction motor imposes a load on the test motor to verify that it functions under rated conditions.

The test environment temperature is 28℃, and the simulated environment temperature is set at 300K. Fig 13 shows the relevant results. Fig 13(a) shows the maximum temperature variation of the motor winding in the motor experiment. After stabilization, the maximum temperature of the winding is 148.63℃, with a temperature rise of 120℃. Fig 13(b) shows the simulation results of the motor temperature field. It has been observed that a 5.76K temperature rise discrepancy exists between the test and simulation results, primarily due to the simplification of the motor winding's end during the simulation process. However, the relative error of their results being less than 5% proves the validity of the simulation model, which further proves the rationality of the simulation model of the double waterway.

Fig 12. Motor test platform

|   (A) |   (B) |
| --- | --- |

Fig 13. Experiment and simulation comparison under rated working conditions. (A) Experiment result, (B) Simulation result.

We have added relevant statements in line 365, page 20 to line 381, page 21.

**Author action:** We updated the manuscript by adding relevant statements. (line 365, page 20 to line 381, page 21).

**Reviewer#2, Concern # 4:** The quantitative effect of the physical parameters (viscosity, specific heat capacity) of different coolants (such as ethylene glycol aqueous solution, deionized water) on the heat dissipation performance is not explained, and only qualitative analysis is performed. It is recommended to clarify the mathematical basis for the selection of coolant.

**Author response:** Thank you very much for your advice. Regarding the selection of coolants, the influence of different physical parameters of coolants on motor performance is mainly reflected in the energy balance equation. In our research, the coolants selected are all types widely used in motors. We have included a parameter table of coolants in the article to have a clearer understanding of the key physical parameters of different coolants. The specific modifications are as follows.

Liquid coolants generally possess a thermal conductivity that is considerably superior to that of air, hence markedly improving the system's heat absorption capability during the heat exchange process. Moreover, during flow, the cooling medium must comply with the principle of conservation of energy. As indicated in Eq (20), fluctuations in the physical characteristics of the cooling medium—namely density, specific heat capacity, and viscosity—directly affect its heat exchange efficiency, consequently influencing the efficacy of its heat dissipation performance.

The choice of coolant substantially influences the motor's cooling efficiency. Four prevalent coolants for automotive engines—water, 30% ethylene glycol solution, 50% ethylene glycol solution, and oil—were chosen to model the temperature distribution of HPDPMSM during the rated functioning of the dual-water cooling system. The coolant specifications are presented in Table 4.

Table 4. Parameters of coolants

| Coolant | Density (kg/m^3^) | Specific heat capacity (J/(kg·K)) | Thermal conductivity (W/(m·K)) | Viscosity coefficient (Kg/(m·s)) |
| --- | --- | --- | --- | --- |
| Water | 998.2 | 4182 | 0.6 | 0.001003 |
| oil | 884 | 2320 | 0.1358 | 0.0014144 |
| 30% ethylene glycol solution | 1020.42 | 3787 | 0.494 | 0.00194 |
| 50% ethylene glycol solution | 1068.75 | 3474 | 0.408 | 0.00298 |

We have added relevant statements in line 383, page 21 to line 395, page 22.

**Author action:** We updated the manuscript by adding relevant statements. (line 383, page 21 to line 395, page 22).

**Reviewer#2, Concern # 5:** Current research has not fully drawn on advanced thermal management theories in the field of energy and power, so the theoretical depth needs to be strengthened. It is recommended to refer to Song et al. (2025) for a review of the heat transfer mechanism of liquid cooling systems to clarify the principles of coordinated optimization of coolant physical parameters and flow channel design (10.1016/j.jpowsour.2025.237227). It is suggested to supplement the collaborative optimization equation of heat transfer coefficient and flow resistance.

**Author response:** Thanks very much for your constructive suggestion. We have referred to the relevant Reference [21] on advanced thermal management theories in the field of energy and power. Meanwhile, we have also supplemented the calculation equation for the heat transfer coefficient and flow resistance. The specific modifications are as follows.

The design of the waterway must account for both fluid flow characteristics and heat exchange efficiency [21]. The heat transfer coefficient and flow resistance are as follows.

The formula for calculating the Reynolds coefficient.

$\mathrm{R}_{\mathrm{e}}=\frac{QD_{e}}{A\nu_{w}}$ (2)

Where, is the equivalent diameters at different positions of the waterway, is cross-sectional area of the waterway, is the flow rate of cooling water in the waterway.

The circumference of the waterway section

$p=2(a+b)$ (4)

Equivalent diameter

$d_{e}=4\frac{A}{p}$ (6)

$h_{f}=\lambda\frac{L_{all}}{d_{e}}\frac{v_{w}^{2}}{2g}$ (10)

Where, is resistance coefficient, is total length of waterways.

Total length of waterways

$L_{all}=\frac{\pi DL}{a+c}$ (11)

Where, D is the diameter of the circle where the center of the waterway is located, L is the motor axial length, c is the interval length between adjacent waterways.

When 2300<$R_{e}$<10^5^, resistance coefficient $\lambda$:

$\lambda=\frac{0.3164}{R_{e}^{0.25}}$ (12)

When 10^5^<$R_{e}$<3×10^6^:

$\lambda=0.0032+\frac{0.221}{R_{e}^{0.237}}$ (13)

The waterways in the motor usually meet: 2300<$R_{e}$<10^5^，so $\lambda=\frac{0.3164}{R_{e}^{0.25}}$

**Calculation of thermal conductivity**

In order to improve the simulation accuracy, the air thermal conductivity inside the motor needs to be calculated.

(23)

Where, is stator inner diameter, is air gap length.

When , the air flow in the air gap is laminar, the effective thermal conductivity is equal to the thermal conductivity of the air.

When , the air flow in the air gap is turbulent, and the formula for calculating the effective thermal conductivity is formula (24).

(24)

The core is typically constructed from thin silicon steel sheets laminated along the axial direction, resulting in pronounced thermal conductivity anisotropy. In the axial direction, the heat conduction process can be modeled as series heat transfer between multiple planar walls. In contrast, in the circumferential and radial directions, it can be approximated as parallel heat transfer between multiple planar walls. The anisotropic thermal conductivity of the core is expressed as follows.

(25)

(26)

Where, is axial thermal conductivity of the iron core, is the circumferential and radial thermal conductivity of the core, is thickness of laminated core, is thickness of insulating medium, is the lamination coefficient of the core, is the thermal conductivity of insulating media, is the thermal conductivity of the laminated core.

Calculation of the equivalent thermal conductivity of slot insulation.

(27)

Where, is the thermal conductivity of equivalent insulation, is the equivalent thickness of each insulating material in the slot， is the thermal conductivity of insulating materials.

[21] Song K, Hou T, Jiang J, Grigoriev SA, Fan F, Qin J, et al. Thermal Management of Liquid-Cooled Proton Exchange Membrane Fuel Cell: A review. Journal of Power Sources. 2025; 648:237227.

We have added relevant statements in line 140, page 8 to line 184, page 10, and line 266, page 15 to line 292, page 16, Ref. [21].

**Author action:** We updated the manuscript by adding relevant statements. (line 140, page 8 to line 184, page 10, and line 266, page 15 to line 292, page 16), and Ref. [21].

**Reviewer#2, Concern # 6:** The dynamic working condition verification method needs to be improved, especially the variable working condition thermal reliability analysis lacks a standardized test framework. The dynamic durability evaluation method of the vehicle power system proposed by Tang et al. (2024) can be transferred to this study, and it is recommended to supplement the high-speed transient temperature rise test (10.1109/TPEL.2024.3502499). It is necessary to quantify the thermal stability threshold of the cooling system during high-speed transient operation of the motor.

**Author response:** Thank you very much for your kind suggestion. We have referred to the relevant Reference [26] on the temperature rise prediction system and have also provided relevant statements. We have respectively added the transient field simulation analysis of the motor at the maximum speed of 16,000rpm and a peak power of 240kW. The simulation results are shown in Fig 17 and 18. The specific modifications are as follows.

**Temperature distributions at different operating conditions**

The operational efficacy and reliability of motors have consistently been critical factors in motor design and development. Particularly at high speeds, EVs experience an augmented electromagnetic strain on the motor, resulting in heightened iron and copper losses, which subsequently elevate the motor's temperature. To guarantee the dependable functioning of EVs within the specified speed range, thermal simulations of the motor are performed under conditions of maximum speed and maximum power operation.

The instantaneous temperature field variations of the motor were studied under stable temperature conditions at rated operating conditions, with the rotational speed increased to the maximum of 16000rpm and a peak power of 240kW. Under the condition of an ambient temperature of 300K, the temperature rise of the motor at its maximum speed is shown in Fig 17. The maximum temperature of the motor winding reaches 438K, showing a temperature rise of 138K, which is 45.23K higher than under rated operating conditions. The maximum temperature of the permanent magnet is 358K, with a temperature rise of 58K, 14.26K higher than the rated operating condition. Fig 18 illustrates the temperature rise of the motor at peak power of 240kW. At maximum power conditions, the motor winding reaches a peak temperature of 442K, with a temperature rise of 142K, while the permanent magnet attains a maximum temperature of 361K, with a temperature rise of 61K. This temperature rise change complies with the maximum operating temperature requirement for the N48UH permanent magnet material, and meets the temperature rise limits allowed for Class H insulation under short-term operating conditions.

The results indicate that, in extreme working conditions, the dual-waterway structure efficiently regulates the motor's internal temperature distribution, preventing overheating and guaranteeing stable performance and long-term reliability at high power and speed levels.

Fig 17. Temperature distributions at maximum speed

Fig 18. Machine temperature distributions at peak power

The aforementioned multi-condition transient simulation results of the motor can furnish data support for the motor dynamic temperature prediction algorithm, thereby facilitating a more comprehensive examination of the algorithm's practicality in assessing motor operating conditions and forecasting motor lifespan [26].

[26] Tang X, Shi L, Li M, Xu S Sun C. Health State Estimation and Long-Term Durability Prediction for Vehicular PEM Fuel Cell Stacks Under Dynamic Operational Conditions. IEEE Transactions on Power Electronics, 2025; 40(3): 4498-4509. doi: 10.1109/TPEL.2024.3502499.

We have added relevant statements in line 432, page 24 to line 461, page 25, and Ref [26].

**Author action:** We updated the manuscript by adding the related statements. (line 432, page 24 to line 461, page 25), and Ref [26].

**Reviewer#2, Concern # 7:** Dynamic operating condition analysis is incomplete. It only analyzes rated operating conditions and does not cover the robustness of the cooling system under high-speed or low-torque transient conditions. It is recommended to add temperature field simulation under variable speed and variable load conditions to verify the adaptability of the system under the full range of operating conditions.

**Author response:** Thanks very much for your comment. We have respectively added the transient field simulation analysis of the motor at the maximum speed of 16,000rpm and a peak power of 240kW. The simulation results are shown in Fig 17 and 18. The specific modifications are as follows.

**Temperature distributions at different operating conditions**

The operational efficacy and reliability of motors have consistently been critical factors in motor design and development. Particularly at elevated speeds, EVs experience an augmented electromagnetic strain on the motor, resulting in heightened iron and copper losses, which subsequently elevate the motor's temperature. To guarantee the dependable functioning of EVs within the specified speed range, thermal simulations of the motor are performed under conditions of maximum speed and maximum power operation.

The instantaneous temperature field variations of the motor were studied under stable temperature conditions at rated operating conditions, with the rotational speed increased to the maximum of 16000rpm and a peak power of 240kW. Under the condition of an ambient temperature of 300K, the temperature rise of the motor at its maximum speed is shown in Fig 17. The maximum temperature of the motor winding reaches 438K, showing a temperature rise of 138K, which is 45.23K higher than under rated operating conditions. The maximum temperature of the permanent magnet is 358K, with a temperature rise of 58K, 14.26K higher than the rated operating condition. Fig 18 illustrates the temperature rise of the motor at peak power of 240 kW. At maximum power conditions, the motor winding reaches a peak temperature of 442K, with a temperature rise of 142K, while the permanent magnet attains a maximum temperature of 361K, with a temperature rise of 61K. This temperature rise change complies with the maximum operating temperature requirement for the N48UH permanent magnet material, and meets the temperature rise limits allowed for Class H insulation under short-term operating conditions.

The results indicate that, in extreme working conditions, the dual-waterway structure efficiently regulates the motor's internal temperature distribution, preventing overheating and guaranteeing stable performance and long-term reliability at elevated power and speed levels.

Fig 17. Temperature distributions at the maximum speed

Fig 18. Machine temperature distributions at peak power

The aforementioned multi-condition transient simulation results of the motor can furnish data support for the motor dynamic temperature prediction algorithm, thereby facilitating a more comprehensive examination of the algorithm's practicality in assessing motor operating conditions and forecasting motor lifespan [26].

We have added relevant statements in line 432, page 24 to line 461, page 25.

**Author action:** We updated the manuscript by adding the related statements. (line 432, page 24 to line 461, page 25).

**Reviewer#2, Concern # 8:** Mechanical reliability analysis is missing. The centrifugal stress of the rotor spiral waterway under high-speed rotation and the durability of the sealing structure are not analyzed, which poses a safety hazard. It is recommended to add ANSYS structural mechanics simulation of the rotor waterway to calculate the strain at the maximum speed and the fatigue life of the seal.

**Author response:** Thank you very much for your kind question. We have added stress simulation analysis of the rotor spiral waterway at the maximum speed. Through this simulation, we verified the stress distribution of the rotor at high speeds and ensured that the maximum stress met the requirements of the material's tensile strength, thereby guaranteeing the reliable operation of the motor under extreme working conditions. The rotor stress distribution is shown in Fig 19. The specific modifications are as follows.

To guarantee that the rotor's cooling structure exhibits substantial tensile strength under high-speed conditions, the stress distribution of the rotor and its corresponding cooling water circuit is examined at the motor's peak operational velocity. Fig 19 illustrates the stress distribution and deformation of the rotor. The maximum stress is 328MPa, with the chosen material B35AH300 demonstrating a tensile strength of 433MPa. The maximum deformation is 0.08mm, which is relatively small and can be negligible.

|   (A) |   (B) |
| --- | --- |

Fig 19. Stress analysis of the rotor at the maximum speed. (A) Stress distribution, (B) Deformation.

We have added relevant statements in line 462, page 25, to line 469, page 25.

**Author action:** We updated the manuscript by adding relevant statements. (line 462, page 25, to line 469, page 25).

**Reviewer#2, Concern # 9:** Pure physical simulation is difficult to cover complex boundary conditions. As shown in [Energies 2024, 17(12), 3050], it is recommended to refer to the Transformer model and explore data-physics fusion simulation to improve the efficiency of temperature field prediction. Dynamic thermal management strategies can be trained using real-time monitoring data, reducing reliance on pure physical simulation.

**Author response:** Thanks very much for your comment. In the motor temperature field simulation section, we realized that complex boundary conditions have a significant impact on the simulation results. The Transformer model explores the fusion simulation method of data and physics to enhance the efficiency of temperature field simulation. This method has made significant contributions to improving simulation accuracy and computing speed. In the text, we have cited relevant Reference [19]. The specific modifications are as follows.

The complicated boundary conditions in the motor temperature field simulation significantly influence the findings. The Transformer model investigates the integration of simulation techniques for data and physics to improve the efficacy of temperature field simulation. This approach has substantially enhanced simulation precision and computational efficiency [19].

[19] Meng X, Mei J, Tang X, Jiang J, Sun C, Song K. The Degradation Prediction of Proton Exchange Membrane Fuel Cell Performance Based on a Transformer Model. Energies. 2024; 17: 3050. doi.org/10.3390/en17123050.

We have added relevant statements in line 80, page 5 to line 84, page 5, and Ref [19].

**Author action:** We updated the manuscript by adding the related statements (line 80, page 5 to line 84, page 5), and Ref [19].
